# A small intestine volvulus caused by strangulation of a mesenteric lipoma: a case report

**DOI:** 10.1186/s13256-017-1232-4

**Published:** 2017-03-13

**Authors:** Yoshihiko Kakiuchi, Hiroaki Mashima, Naoto Hori, Hirotoshi Takashima

**Affiliations:** Department of Gastroenterological Surgery, Syobara Red Cross Hospital, 2-7-10, Nishihonmachi, Shobara city, Hiroshima 727-0013 Japan

**Keywords:** Mesenteric lipoma, Strangulation, Laparoscopic surgery, Case report

## Abstract

**Background:**

An emergency department encounters a variety of cases, including rare cases of the strangulation of a mesenteric lipoma by the greater omentum band.

**Case presentation:**

A 67-year-old Japanese man presented with nausea, vomiting, and upper abdominal pain. There were no abnormalities detected by routine blood tests other than a slight rise in his white cell count. A contrast-enhanced computed tomography scan of his abdomen revealed a dilated intestine, a small intestine volvulus, and a well-capsulated homogeneous mass. He was suspected of having a small intestine volvulus that was affected by a mesenteric lipoma; therefore, single-port laparoscopic surgery was performed. Laparoscopy revealed a small intestine volvulus secondary to the strangulation of a mesenteric lipoma. The band and tumor were removed. He had no postoperative complications and was discharged on postoperative day 6.

**Conclusions:**

Although this case was an emergency, it showed that single-port laparoscopic surgery can be a safe, useful, and efficacious procedure.

## Background

Lipomas are benign neoplasms of adipose tissue that can occur almost anywhere. Mesenteric lipomas are uncommon [1], but strangulation of mesenteric lipomas is exceptionally rare. Here, we describe a case of a small intestine volvulus caused by strangulation of a mesenteric lipoma by the greater omentum band, which was successfully managed by performing single-port laparoscopic surgery.

## Case presentation

**Fig. 1:**
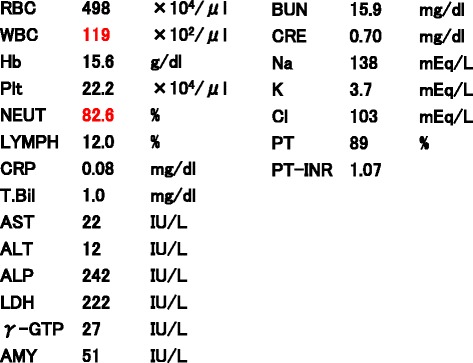
Results of laboratory findings. *ALP* alkaline phosphatase, *ALT* alanine aminotransferase, *AMY* amylase, *AST* aspartate aminotransferase, *BUN* blood urea nitrogen, *Cl* chlorine, *CRE* creatinine, *CRP* C-reactive protein, *γ-GTP* gamma-glutamyl transpeptidase, *Hb* hemoglobin, *K* potassium, *LDH* lactate dehydrogenase, *LYMPH* lymphocytes, *Na* sodium, *NEUT* neutrophils, *Plt* platelets, *PT* prothrombin time, *PT-INR* prothrombin time-international normalized ratio, *RBC* red blood cells, *T.Bil* total bilirubin, *WBC* white blood cells

**Fig. 2:**
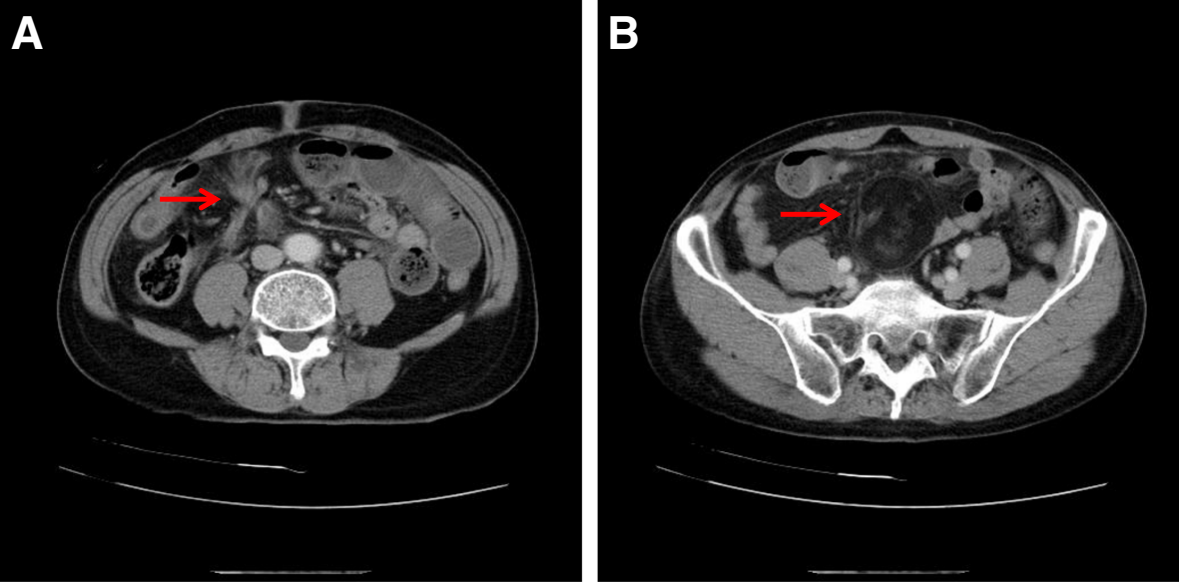
A contrast-enhanced computed tomography scan of the abdomen. **a** The twisted small intestine (*arrow*). **b** A well-capsulated homogeneous mass (*arrow*)

A 67-year-old Japanese man presented with a 1-day history of nausea, vomiting, and upper abdominal pain. He described that these symptoms had occurred intermittently for several years, and had previously alleviated naturally; however, in this instance, they did not alleviate. Although he used to take medication for hypertension, he had no surgical, family, social, or environmental history. A physical examination revealed only a slightly distended abdomen. A neurological examination showed no abnormality. His vital signs on admission were: blood pressure 152/96 mmHg, heart rate 54 beats per minute (bpm), and body temperature 36.5 °C. A laboratory investigation revealed a white blood cell count of 11,900 cells/μL comprising 82.6 % segmented neutrophils (Fig. 1). Abdominal radiography revealed a prominently dilated small intestine with some air–fluid interfaces. A contrast-enhanced computed tomography scan of his abdomen revealed a dilated intestine, a small intestine volvulus, and a well-capsulated homogeneous mass (Fig. 2). Volvulus of the intestine, by the twisted appearance of the main mesenteric vessels in their root, was discovered. He was diagnosed as having a small intestine volvulus that was affected by a lipoma. Single-port laparoscopic surgery was performed.

**Fig. 3:**
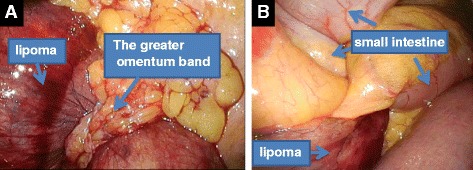
Operation views. **a** The lipoma strangulated by the greater omentum band. **b** The twisted small intestine and lipoma

**Fig. 4:**
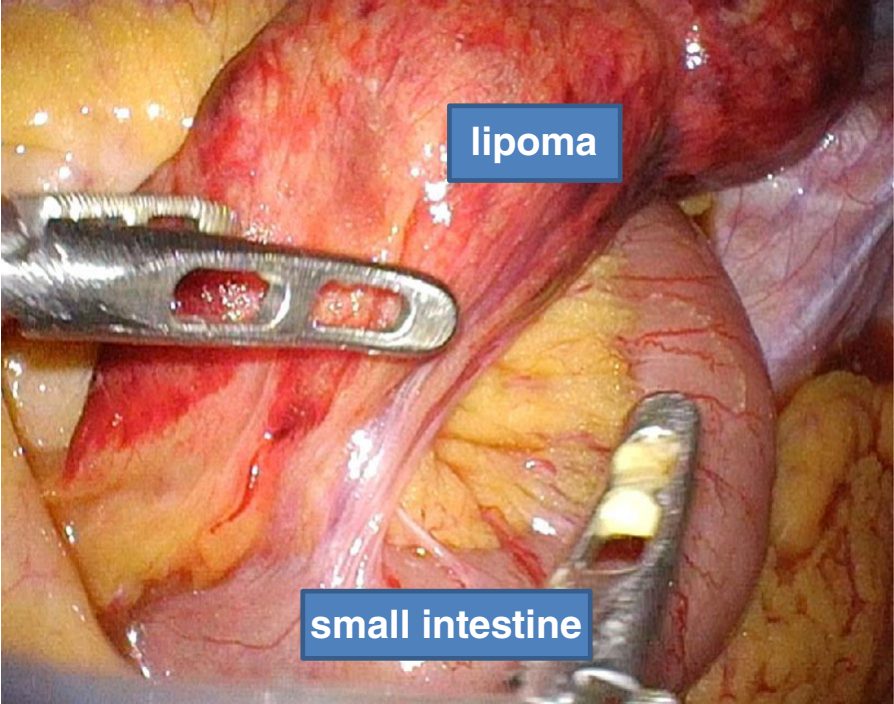
The tumor is loose after surgical removal of the greater omentum band

**Fig. 5:**
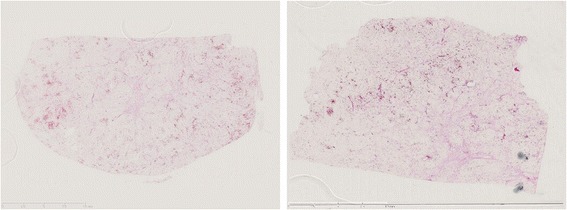
Mature adipocyte-like normal adipose tissue

He was placed under general anesthesia in the supine position. A 2 cm incision was created in his umbilical region, and covered with a wrapping protector. Three 5 mm laparoscopic cannulae were introduced through the access channels of the multi-trocar device at the 3, 7, and 11 o’clock positions. Following initial abdominal exploration, a twisted small intestine and congested tissue were found. The congested tissue was caused by the greater omentum band (Fig. 3); therefore, the band was removed. After having resolved the twisting, we determined that the congested tissue was a mesenteric lipoma. A mesenteric lipoma was identified in the terminal ileum approximately 20 cm proximal to the ileocecal valve; it was a well-capsulated, 10×9 cm, smooth, yellowish mass originating from the small mesentery. Because this tumor occurred from the mesentery (Fig. 4), resection of his small intestine was unnecessary. Laparoscopic resection of the origin of this tumor was performed using an ultrasonic coagulation device. The umbilical incision was extended to 5 cm and the tumor was retrieved through the incision using a bag without injury. The tumor was removed *en bloc*. Surgical blood loss was 20 ml, and operative time was 92 minutes. Our patient had no postoperative complications and was discharged on postoperative day 6. A histopathological examination revealed that the tumor was composed of mature adipocyte-like normal adipose tissue, confirming that it was a lipoma (Fig. 5). There was no recurrence or complications 1-year post-operation.

## Discussion

A lipoma is a benign tumor of mature adipocytes. A lipoma can occur almost anywhere in the trunk, extremities, or even intraperitoneally, which is extremely rare with a small overall malignant potential [2]. The differential diagnosis is liposarcoma, which has a high recurrence rate. However, as abdominal ultrasonography and magnetic resonance imaging may show typical findings [3–5], it is difficult to diagnose exactly in an emergency case. In the case of an emergency surgery, like the one presented here, complete removal of the mass is important.

A lipoma is often accidentally detected secondary to other symptoms. The symptoms in many cases are abdominal swelling, abdominal pain, or mass palpation [6]. The symptoms in our case were nausea, vomiting, and upper abdominal pain, which were different from typical symptoms and it was thought that they were caused by a small intestine volvulus that was affected by a mesenteric lipoma. Strangulation of the mesenteric lipoma due to the greater omentum band was recognized as a cause of the volvulus during laparoscopic surgery.

The tumor was identified; however, the nature of its involvement in the twisting was not known. Therefore, the first operation was performed to resolve the twisting. Single-port laparoscopic surgery was chosen because the intestinal volvulus was believed to be corrected and it was expected that the surgery would not be very challenging to perform. In the case of the cancellation of the volvulus, the enucleation of the tumor, and the partial resection of the small bowel, laparoscopic surgery, specifically single port, is a very good adaptation. In the case of a small open surgery, we may overlook a tumor that is in other locations because we cannot observe it properly. In addition, pain reduction and early resumption of oral food intake are more likely to lead to shortening of the hospital stay. However, it is preferable for an operation to be performed by an expert surgeon because it is necessary to resolve the twisting and observe the entire small intestine at which time complications such as intestinal tract damage may be caused by the operation of forceps. If the operation is difficult, the addition of a port or a small laparotomy should be performed. We must remember that safety is the topmost priority.

## Conclusions

The strangulation of a mesenteric lipoma by the greater omentum band is rare. To the best of our knowledge, this is the first report of not only the pathophysiology but also the treatment by single-port laparoscopic surgery.

## References

[1] Ilhan H, Toker B, Isiksoy S, Koku N, Pasaoglu O. Giant mesenteric lipoma. J Pediatr Surg. 1999;34:639–40. 10.1016/s0022-3468(99)90094-5 10235343

[2] Sheen AJ, Drake I, George PP. A small bowel volvulus caused by a mesenteric lipoma: report of a case. Surg Today. 2003;33:617–9. 10.1007/s00595-003-2533-3 12884101

[3] Chehade HH, Zbibo RH, Nasreddine W, Abtar HK. Large ileocecal submucosal lipoma presenting as hematochezia, a case report and review of literature. Int J Surg Case Rep. 2015;10:1–4. 10.1016/j.ijscr.2015.03.007 PMC4429855 25770697

[4] Kang B, Zhang Q, Shang D, Ni Q, Muhammad F, Hou L, Cui W. Resolution of intussusception after spontaneous expulsion of an ileal lipoma per rectum: a case report and literature review. World J Surg Oncol. 2014;12:143. 10.1186/1477-7819-12-143 PMC4038827 24884620

[5] Friedman AC, Hartman DS, Sherman J, Lautin EM, Goldman M. Computed tomography of abdominal fatty masses. Radiology. 1981;139:415–29. 10.1148/radiology.139.2.7220888 7220888

[6] Cha JM, Lee JI, Joo KR, Choe JW, Jung SW, Shin HP, Kim HC, Lee SH, Lim SJ. Giant mesenteric lipoma as an unusual cause of abdominal pain: a case report and a review of the literature. J Korean Med Sci. 2009;24:333–6. 10.3346/jkms.2009.24.2.333 PMC2672139 19399281

